# Social work after stroke: identifying demand for support by recording stroke patients’ and carers’ needs in different phases after stroke

**DOI:** 10.1186/s12883-016-0626-z

**Published:** 2016-07-20

**Authors:** Inken Padberg, Petra Knispel, Susanne Zöllner, Meike Sieveking, Alice Schneider, Jens Steinbrink, Peter U. Heuschmann, Ian Wellwood, Andreas Meisel

**Affiliations:** Center for Stroke Research Berlin (CSB), Charité University Medicine, Berlin, Germany; Berliner Schlaganfall-Allianz e.V., Berlin, Germany; Institute of Clinical Epidemiology and Biometry, University of Würzburg, Würzburg, Germany; Clinical Trial Center Würzburg, University Hospital Würzburg, Würzburg, Germany; Institute of Public Health, University of Cambridge, Cambridge, UK; Neurocure Clinical Research Center, Department of Neurology, Charité University Medicine, Berlin, Germany; Research Group “Clinical Epidemiology and Health Services in Stroke“(CEHRiS), Center for Stroke Research Berlin (CSB), Charité University Medicine, Charitéplatz 1, 10117 Berlin, Germany; Comprehensive Heart Failure Center, University of Würzburg, Würzburg, Germany

**Keywords:** Social support, Stroke, Rehabilitation, Social work, Patient-centered care

## Abstract

**Background:**

Previous studies examining social work interventions in stroke often lack information on content, methods and timing over different phases of care including acute hospital, rehabilitation and out-patient care. This limits our ability to evaluate the impact of social work in multidisciplinary stroke care.

We aimed to quantify social-work-related support in stroke patients and their carers in terms of timing and content, depending on the different phases of stroke care.

**Methods:**

We prospectively collected and evaluated data derived from a specialized “Stroke-Service-Point” (SSP); a “drop in” center and non-medical stroke assistance service, staffed by social workers and available to all stroke patients, their carers and members of the public in the metropolitan region of Berlin, Germany.

**Results:**

Enquiries from 257 consenting participants consulting the SSP between March 2010 and April 2012 related to out-patient and in-patient services, therapeutic services, medical questions, medical rehabilitation, self-help groups and questions around obtaining benefits. Frequency of enquiries for different topics depended on whether patients were located in an in-patient or out-patient setting. The majority of contacts involved information provision. While the proportion of male and female patients with stroke was similar, about two thirds of the carers contacting the SSP were female.

**Conclusion:**

The social-work-related services provided by a specialized center in a German metropolitan area were diverse in terms of topic and timing depending on the phase of stroke care. Targeting the timing of interventions might be important to increase the impact of social work on patient’s outcome.

**Electronic supplementary material:**

The online version of this article (doi:10.1186/s12883-016-0626-z) contains supplementary material, which is available to authorized users.

## Background

### Social work in stroke

Providing care for survivors of stroke can be complex, requiring a combination of medical, nursing, therapeutic and social interventions [1]. Social work covers diverse aspects of care such as counselling, liaison with other services, provision of information (eg advice on how to obtain benefits, contact details of medical doctors and self-help groups) and help with arranging housekeeping or nursing interventions (including assistance with personal care and medication for secondary prevention) [1]. Social workers work with stroke patients in the out-patient phase of the disease but are also members of the multi-disciplinary teams taking care of stroke patients in acute hospitals and during in-patient rehabilitation (in-patient phase) [1, 2]. Often social work commences during the acute phase of stroke and is required long after discharge from hospital.

With such a broad scope, it can be challenging to precisely define and describe social work interventions in studies evaluating multidisciplinary care after stroke. In its broadest sense, social work aims to help the patient, their family and the acute or rehabilitation team to reach individually determined goals. Social workers assist stroke patients in the process of adjustment to disability and where possible facilitate the patient’s return to the community at the highest possible functional, social and economic level [3].

### Previous studies on social work in stroke

The literature on interventions relevant to social work in stroke is diverse and covers many aspects of health and social care. For example, provision of information has been shown in a Cochrane systematic review to improve patients’ and carers’ knowledge of stroke and aspects of patient satisfaction [4].

In the out-patient phase of stroke, combined delivery of education and counselling by social workers has been described as important for patient adjustment [5].

Long term adjustment to stroke can be particularly challenging, with social inactivity and symptoms of depression occurring during the chronic phase of the disease [6]. McCarthy et al. underline the important role of the social worker in addressing post-stroke depression after discharge [7].

The need and demand for different forms of assistance by caregivers and patients may change over time. For caregivers, the literature suggests that interventions should be tailored to individual caregiver’s needs and include psychotherapeutic and psycho-educational components [8]. Thus far, few studies have investigated the most appropriate timing of provision of such support with the exception of a recent pilot study “Timing it Right Stroke Family Support Program” which considered five phases of stroke; acute transmission, medical stabilization, preparation for discharge home, first few month at home and longer term adjustment [9].

In a systematic review on stroke liaison workers (multifaceted services that were also termed “social work” [10], “specialized nurse support” [11], “stroke family care worker” [12], or “stroke family support organizer” [13]) published after our study commenced, the reviewers evaluated 16 studies (involving 4759 participants) providing education, emotional and social support (including counselling) [1, 10–21]. The authors concluded that overall, there was “no evidence for the effectiveness of this multifaceted intervention in improving outcomes for all groups of patients or carers”. However these services associated with stroke liaison workers covered aspects of stroke care often provided after discharge from hospital with about half the published studies including patients from out-patient settings [10, 15, 19–21]. Consequently, the role of social workers as part of the multidisciplinary teams caring for patients during the acute or rehabilitation in-patient phases of stroke care was not addressed in detail.

The review called for further research in the area.

### Need for further research/gaps in knowledge

Our prospective observational study aimed to quantify, describe and explore social work services requested by and provided to patients and carers contacting an independent “Stroke- Service-Point” (SSP); a form of freely available “drop in” centre and non-medical stroke assistance service staffed by trained social workers in Berlin, Germany. The SSP, offers information, advice and individualized counselling on stroke risk factors, health promoting lifestyle, importance of medication compliance and interactions with healthcare services with similarities to the stroke liaison worker [1]. The SSP is available to all patients and caregivers allowing us to look for differences between in patient and out-patient setting. We were particularly interested in differences in requests for and provision of social work across these different phases of stroke care; to allow future social work services to be targeted at the most appropriate time.

## Methods

### Data collection

We collected data on the SSP which is provided in collaboration with the Berlin Stroke Alliance (BSA; www.schlaganfallcentrum.de/en/patient-information/berlin-stroke-alliance/); a network of 39 facilities (hospitals, rehabilitation clinics, nursing homes, out-patient care facilities), support groups and non-profit associations in Berlin and Brandenburg. The SSP is located on a hospital campus in the city centre and the social workers do not undertake home visits. The service was established in addition to routinely available social work infrastructures in the hospital and rehabilitation facilities. As such, it represents an additional opportunity, without the need for medical referral, for patients and their carers to obtain information and support after stroke.

Any person (people with stroke, their carers and health care professionals) contacting the SSP was eligible and invited to participate in the study when they visited, telephoned, or wrote to the SSP. The SSP social workers systematically collected information about the social work related enquiries they received in terms of who made the enquiry, the topics of interest, the location, age, sex and home district of the person with stroke, length of time since stroke onset and the method of contact (in person, by phone, via website). Data were collected prospectively on a structured form. Social work services were recorded using 11 categories (provision of in-patient services, medical rehabilitation, out-patient assistance, therapeutic and preventive services, nursing care, partly residential services, assistance with reintegration, vocational advice (return to working life), medical questions, questions around obtaining benefits, and “other” topics). Data on the duration, number and content of the topics discussed and any social work actions taken during contact with the SSP were also recorded. Finally, data summarising the outcome of the contact was recorded.

Details of data collection, ethics consent and permission and design/development of the questionnaire are provided in the Additional file 1 and in Additional file 2.

### Data analysis

Descriptive statistics were used to analyse the data with SPSS 22.0 software. Data are presented as counts and percentages of enquiries by patients and carers about specific subjects. Differences in the stroke-related topics of interest discussed by contacts in relation to patients in in-patient or out-patient settings were calculated using the chi-square test.

## Results

### Persons making contact to the SSP

Between the beginning of March 2010 and end of April 2012, a total of 1228 contacts were made to the SSP of which 257 people participated in the study (response rate 20.9 %). The people contacting the SSP were: stroke patients (*n* = 71; 27.6 %); relatives involved in taking care of the patients, (*n* = 157; 61.1 %) and friends or healthcare professionals (*n* = 10; 3.8 %) (See Table 1). Of the relatives involved in patient care, 105 (66.9 %) were female, 35 (22.3 %) were male and in 17 cases (10.8 %) sex was not recorded. Around half (50.3 %) the people contacting the SSP were aged 45 to 64 years old (50.3 %), 44 (28.8 %) were 65 years and older and 32 (20.9 %) younger than 45 years.

**Table 1:** Overview of participant and patient characteristics

Person making contact with SSP		Patient	Care taking relative	Friend or health care professional	Not recorded	Total
Age of patient (years)	<45	10	8	1	4	23
	45–64	25	56	6	3	90
	>/=65	36	93	3	12	144
Phase of stroke	acute hospital	0	26	0	3	29
	rehabilitation hospital	0	46	2	0	48
	in nursing care	1	16	1	2	20
	out-patient	70	69	7	14	160
Method of contact	telephone	30	69	5	3	107
	face to face	41	81	5	16	143
	written	0	7	0	0	7
Sex of patient	male	36	91	6	10	143
	female	34	58	1	9	102
	not recorded	1	8	3	0	12
Time after stroke (month)^a^	<6	18	91	3	3	115
	>6	38	57	6	10	111
	not recorded	5	9	1	6	21

^a^An additional 10 patients were at high risk of stroke due to medical and familial risk factors but no stroke had taken place. Interest in this group was focused on medical and therapeutic questions. Including or excluding these patients from general analysis did not significantly change the results (Table 2)

### Stroke patients

Table 1 presents details of 257 stroke patients (*n* =143 (55.6 %) male, 102 (39.7 %) female, 12 (4.7 %) missing). Most patients (*n* = 144; (56.1 %)) were 65 years and older. The stroke had occurred within six months in 115 (44.8 %) patients and longer than this in 111 (43.2 %) patients. Patients presented from all 12 districts of Berlin (range 4.7–11.7 % per district).

### Type of contacts

**Fig. 1:**
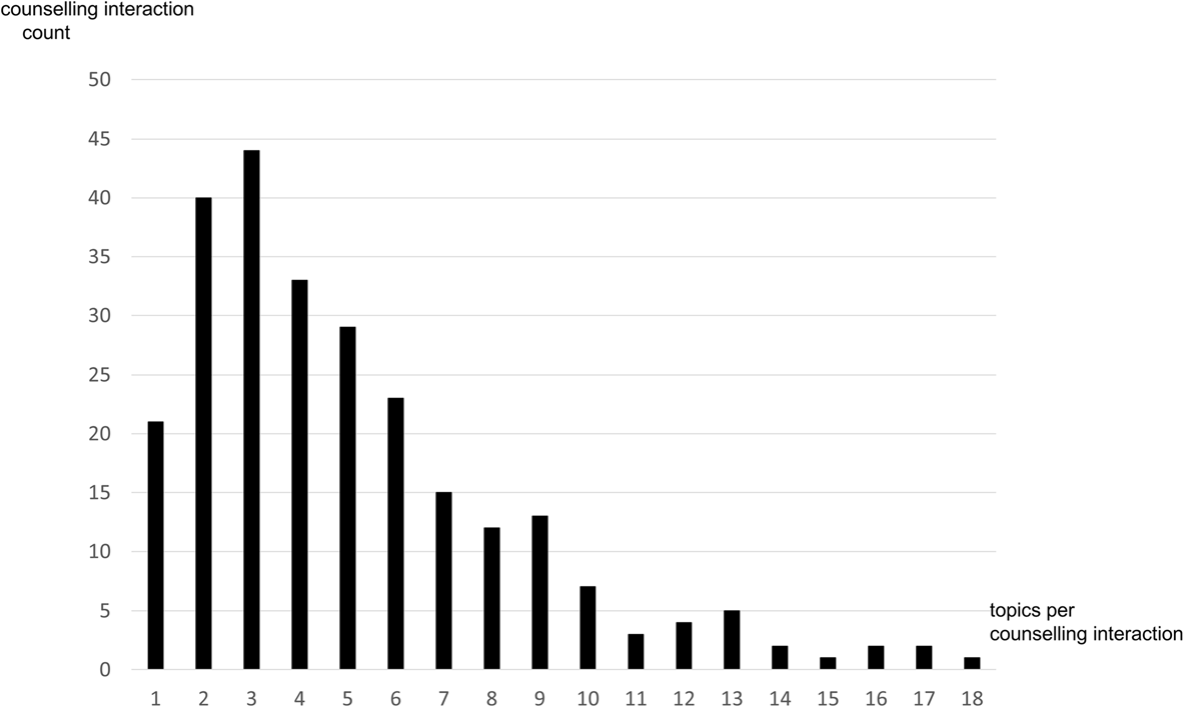
Numbers of topics in individual counselling interaction with the SSP. Displayed is the distribution of the cumulated number of topics addressed in single sessions. All subjects were included in the analysis

Of the 257 study participants 143 (55.6 %) contacts were face to face, 107 (41.6 %) by telephone and 7 (2.7 %) were written (e-mail) (Table 1),(compared to 24.7, 64.7 and 10.6 % respectively in all 1228 contacts made to the SSP during the study period). The mean duration of a contact was 49 min (range 10–120 min). Most participants (*n* = 218; (84.8 %)) made only one contact whilst 30 (11.7 %) made two contacts with the SSP. The number of topics addressed per contact varied from 1 to 18 with most addressing 1–3 topics (Fig. 1).

### Social work actions

Social workers described the actions they took in managing requests for assistance. Here the vast majority, 246 (95.7 %) involved the provision of information and an explanatory discussion. In 75 cases (29.2 %) the contact resulted in referral to another service provider, in 50 cases (19.5 %) in provision of aids, equipment and home modifications. In 4 cases (1.6 %) action involved providing assistance with social benefit applications (eg pension initiated after 18 month of inability to work, or application for vocational integration), 3 (1.2 %) cases involved handling appeals (eg rejection of application for an identity card for people with marked activity restriction (to confirm status with employees, authorities etc. or for nursing care insurance) and 6 (2.3 %) contacts related to complaints.

### Services provided to stroke patients and carers

Topics of most interest to SSP contacts were out-patient services, (*n* = 102; 39.7 %), in-patient services, (*n* = 50; 19.5 %), questions around medical treatment (*n* = 77; 30 %), medical rehabilitation (*n* = 89; 34.6 %), questions around obtaining benefits (*n* = 145; 56.4 %), self-help groups (*n* = 95; 37.0 %) and therapeutic or preventive services (*n* = 140; 54.5 %). Overall, there was interest in at least one topic around therapeutic and preventive services in over 60 % of patients younger than 65 years and almost 50 % in cases older than 65 years. Specific subcategories for therapeutic and preventive services of interest were occupational therapy (*n* = 70; 27.2 %), speech therapy (*n* = 65; 25.3 %), physiotherapy (*n* = 87; 33.9 %) and neuropsychology (*n* = 64; 24.9 %). Questions around medical treatment included subcategories related to finding a medical practitioner specialized in out-patient care for stroke (*n* = 45; 17.5 %), secondary prevention (*n* = 30; 11.7 %) and acute or rehabilitation treatment (*n* = 31; 12.1 %). With regard to topics and subcategories around out-patient services, 41 (15.9 %) participants were interested in out-patient nursing care (including general home care, palliative care, family care and short term home care), 57 (22.2 %) participants were interested in home adaptation/aids (including changes around the house, emergency house calls and other aids at home) and 42 (16.3 %) were interested in help with mobility/transport (including driving services for recreation or provision of companions for people with decreased mobility). With regard to medical rehabilitation, 40 (15.6 %) participants were interested in more information about out-patient rehabilitation, 37 (14.4 %) in in-patient rehabilitation and 36 (14.0 %) in services related to long term in-patient care. Subcategories of benefits were summarized as this information is specific to the German health care system.

### Services provided in association with the stage of stroke care

**Fig. 2:**
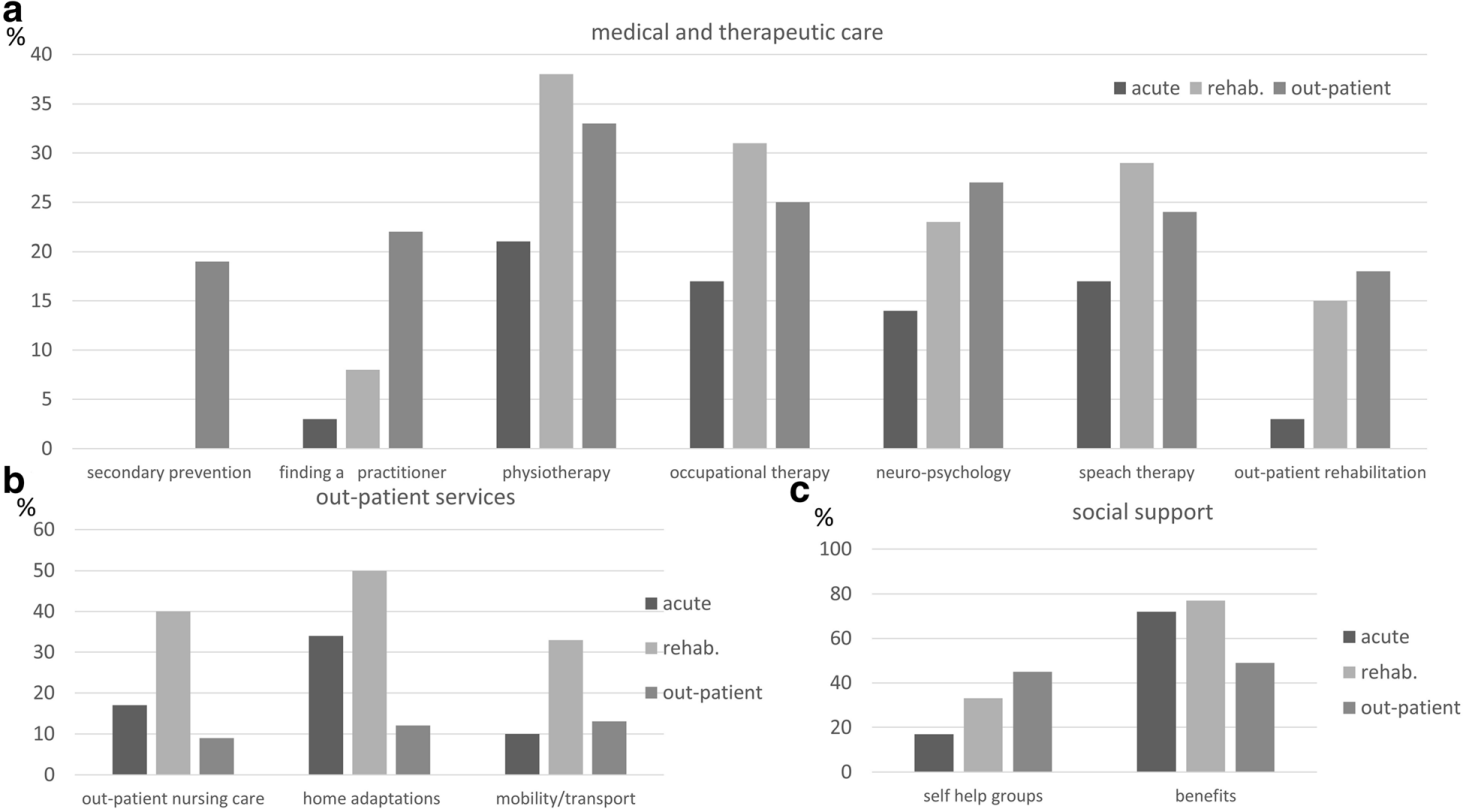
Frequency of requested topics in counselling in relation to the place of residence. The most relevant topics (>10 % of total) of interest to clients of the SSP are shown in relation to the different phases of stroke (in % of total). At the time of the interaction, 29 (11.3 %) patients were still being treated in a hospital (acute), 48 (18.7 %) in a rehabilitation clinic (rehab.), 160 (62.3 %) were living at home (out-patient stroke care). Topics are shown in relation to medical and therapeutic stroke care (including medical information, out-patient rehabilitation and therapeutic and preventive services) **a**, out-patient services **b** and social support **c**

The frequency of enquiry about topics by patients and carers differed depending on the location (hospital, rehabilitation clinic or at home) of the person with stroke (Fig. 2). For some topics associated with out-patient stroke care such as obtaining homecare or home adaptations, there was significantly more interest when the person with stroke was still in an in-patient setting (Table 2, Fig. 2). In contrast, for topics relating to medical problems such as finding a specialized out-patient practitioner and for questions around secondary prevention, there was significantly more interest when patients had been discharged from hospital (Table 2, Fig. 2). The same was true for self-help groups. Levels of interest in therapeutic and preventive topics were similar between in-patient or out-patient settings (Fig. 2). Information on social benefits was of interest to 75 % of all cases where the patient was still an in-patient and to 50 % of cases where the patients were out-patients.

**Table 2:** Comparison of differences in social work services provided by Stroke Service Point according to location of patient with stroke

		N total = 237 (excluding patients in nursing care)				
		n (service provided)	n (no service provided)	n (service provided)	n (no service provided)	*p*-value
	Topics of special interest	Acute/rehabilitation hospital		Out-patient		
Out-patient services	out-patient nursing care	24	53	15	145	2,3E-05
	home adjustments	34	43	19	141	2,3E-08
	transport	19	58	20	140	0,0181
Medical information	secondary prevention	0	77	30	130	4,8E-05
	finding specialized out-patient practitioner	5	72	25	135	0,003
Other	self help groups	21	56	72	88	0,009

When patients were still in an acute hospital or rehabilitation hospital, contact to the SSP was done by relatives involved in providing care. Differences seen in the previous analysis might be primarily caused by differences in the patients’ vs. the caregivers’ perception of need and not by a general difference between in-patient and out-patient expression of need. To exclude this possibility, we repeated the analysis involving only the caregiving relatives but not the patients themselves as the contact person. Except for the topic of searching for a specialized out-patient practitioner, where the difference in services provided decreased when excluding the patients that made direct contact with the SSP in the out-patient phase, the results were similar..

## Discussion

Our observational study revealed: The topics of greatest interest to stroke patients and carers contacting the SSP were: out-patient and in-patient services, medical treatment, medical rehabilitation, therapeutic or preventive services, questions around obtaining benefits and information on self-help groups. Social work service enquiries varied significantly over the different phases of stroke care. Even when patients were in hospital, carers often wanted advice, on topics mainly related to the out-patient setting such as obtaining home adaptations. The vast majority of contacts involved the provision of information or an explanation of information obtained elsewhere. Although the proportion of men and women with stroke was approximately equal, the majority (about two thirds) of carers contacting the SSP were women.

Contact to the SSP was often made by a relative involved in taking care of the patient. This was especially true when the patient still was in the acute or rehabilitation hospital, presumably because it was harder for patients to make contact whilst still in hospital. The fact that the majority of carers contacting the SSP were women could indicate that the burden of care lies predominantly with women, or that woman were more likely to seek information.

Social work staff in the SSP offered a range of services including social and emotional support, provision of advice and information as well as liaison with other services similar to the function of stroke liaison workers as described by Ellis et al. [1]. The role of the stroke liaison worker in this context has been mainly considered in the out-patient setting.

As social work is important in all phases of stroke care, we examined services provided according to the topics covered in the interventions across acute, rehabilitation and out-patient stroke care. We found service provision changed depending on the location of patients with stroke at SSP contact. To our knowledge the SSP study is the first to consider the timing of social work interventions to be important for patients as well as caregivers in stroke healthcare.

Our data show that interest in topics centred on the living situation of patients at home (home adaptations, transport, out-patient nursing care) was higher when the patient was still in hospital. In contrast, interest in information on topics such as self-help groups, secondary prevention and finding a specialized out-patient practitioner in our study was much higher in the out-patient setting. This finding suggests that already in the acute course of stroke, carers and patients are aware of the importance of this issue. However, it may also indicate that patients and carers who are unable to receive appropriate information during institutional acute or rehabilitation care, stopped searching or never started to search for better solutions eg in the context of home adaptations, but instead adjusted to their living situation at home. For patients and relatives, a prolonged application process for additional benefits or a medical re-evaluation may be stressful and they may find it easier to accept their circumstances. Consequently, studies that only include patients following discharge may fail to identify needs that could have been negated through earlier social work intervention.

Previous studies report that stroke patients were often unaware of benefits and services available to them and whether or not they could access services again after they had been discharged [22]. Patients furthermore reported struggling with bureaucratic procedures related to obtaining health and social services [22]. A quantitative study of community stroke care in Seattle, showed sub-optimal home care was associated with risk of depression in caregivers, family dysfunction and below average knowledge about stroke care. Social work for people with stroke might therefore include working on family function and caregiver well-being in addition to knowledge about stroke [5] and be important even before patients return to the community. Our study also indicates that patients and caregivers were interested in planning future practical aspects of care such as home aids/adaptations during the acute and rehabilitation phase of the disease.

A previous study from the UK showed that stroke patients were mostly satisfied with information and advice they received with regard to lifestyle and health promotion issues, incontinence and current treatment [23]. However patients were dissatisfied with information on stroke disease in general, its effects, available services and legal and financial advice [23]. Our study confirms that information on financial affairs (benefits) is also an important topic in the German healthcare system during all phases of stroke care, but particularly in the acute and rehabilitation hospital settings.

In a cross-sectional survey conducted in the UK using a population based stroke register and national general practice research framework, self-reported long term need was assessed in patients 1–5 years after stroke [24]. This study found persistent long term social needs in areas such as benefit advice, information on stroke, family and partner relationships, employment and increased expenses [24].

Results from the above mentioned studies and our study suggest that common themes (such as lack of information on stroke, stroke care in general, or information on benefits) can arise in different contexts and in very different health care systems.

Apart from information on financial affairs and self-help groups, one of the strongest requests in our study was for further information about therapeutic options. This might indicate difficulties in finding adequate treatment in the rehabilitation and out-patient stroke care. Also a qualitative study in the UK [25] explored stroke survivors’ and care-givers’ experiences and views of the rehabilitation assessment process, finding a demand for more information about the purpose of assessments as well as regular, and objective feedback about the patients’ progress [25] In a prospective observational study of the rehabilitation process after stroke in Stockholm, Sweden more severe stroke and poor self-rated recovery actually predicted unfulfilled rehabilitation needs, while limited participation and limited coping capacity predicted dissatisfaction with care at 12 months after stroke [26].

Requests for information on therapeutic interventions might reflect such unfulfilled rehabilitation needs or dissatisfaction with care.

### Limitations

Similar to previous studies there is a risk that the topics we identified reflected not only items raised by patients or care givers, but potentially those considered important by the social worker. However, in our study the patients and care givers were self-motivated and actively sought contact with the SSP to discuss specific topics and we feel these topics were adequately captured rather than simply describing a standard package of advice or care.

Data were generated in Germany where the healthcare system has a number of unique features that might limit generalizability to other health care systems. Every citizen in Germany should have access to medical treatment, rehabilitation and support. However, getting full access to all support, in particular for people with chronic disabling disorders is complex. Thus, it is often difficult for patients and relatives to know the kind of support to which they are entitled and how it can be accessed and funded. Furthermore, a social worker is assigned during acute and rehabilitation care only when counselling on social law issues and further social support is considered as necessary by a medical doctor. Having investigated data from 257 participants over a two year period in an urban area we have only a snapshot of the social care service in the German healthcare system. Our findings are further limited by the fact that we were not able to follow up participants or examine aspects such as cost, uptake of services, satisfaction, short or long-term outcomes. Furthermore the service is specifically designed to offer additional support and need not be offered to all patients. Consequently the selected subset of patients and carers may not be representative of the whole stroke population and the SSP may also not be the primary provider of social work services for many respondents. The rather low response rate in our study might be due to difficulties in recruiting potential participants who made contact to the SSP by email or telephone. Such contact is less personal and individuals may be less inclined to participate. Variation in the type of contact, the low response rate, the fact that the SSP interaction was not always the first interaction with a social work provider and the different data sources arguably make interpretation of the data difficult. We did however achieve consistency with regard to recording the topics that were of interest to the patients and caregivers by using the same structured questionnaire. Although these limitations might restrict the generalizability and interpretation of some of our findings, our data quantify social services requested by stroke patients and carers along all phases of stroke care at an independent stroke service point.

## Conclusion

Our data suggest considerable differences in social work services provided depending on the phase of post-stroke care. Increased knowledge of needs for social work interventions depending on the phase of stroke care might contribute to a more timely and tailored approach which may improve care for stroke patients and their relatives. We identified that provision of benefits, therapeutic and preventive services and care aids for the out-patient setting were important topics to be covered in social work interventions. Future research might target the impact of precise timing of the provision of information on the effectiveness of different social work interventions in more detail.

## Abbreviation

SSP, stroke-service-point

## Additional files

Additional file 1: Recruitment and verbal consent. Description of recruitment as well as obtaining and documentation of consent. Data management. Description of data management and generation of the questionnaire. (DOCX 18 kb) Additional file 2: Table S1. Categories for description of social work service. A comparison of general description for social work services in the health care system with a stroke specific expanded service description used to generate the questionnaire used in the current study. (DOCX 12 kb)

## References

[1] Ellis G, Mant J, Langhorne P, Dennis M, Winner S. Stroke liaison workers for stroke patients and carers: an individual patient data meta-analysis. Cochrane Database Syst Rev. 2010;2010(5):1–101. 10.1002/14651858.CD005066.pub2 PMC6464840 20464736

[2] Rizzo VM (2006). Social Work Support Services for Stroke Patients. Soc Work Health Care.

[3] Ue B. Social work and the stroke patient. Clin Orthop Relat Res. 1978;1978(131):101–103. 657605

[4] Forster A, Brown L, Smith J, House A, Knapp P, Wright JJ, Young J. Information provision for stroke patients and their caregivers. Cochrane Database Syst Rev. 2012;2012(11):1-131.

[5] Evans RL, Matlock AL, Bishop DS, Stranahan S, Pederson C (1988). Family intervention after stroke: does counseling or education help?. Stroke.

[6] Mutai H, Fukarawa T, Araki K, Misawa K, Hanihara T. Long-term outcome in stroke survivors after discharge from a convalescent rehabilitation ward. Psychiatry Clin Neurosci. 2013;67(6):434–440. 10.1111/pcn.12075 23941061

[7] McCarthy MJ, Powers LE, Lyons KS. Poststroke depression: social workers’ role in addressing an underrecognized psychological problem for couples who have experienced stroke. Health Soc Work. 2011;36(2):139–148. 10.1093/hsw/36.2.139 21661303

[8] Sörensen S, Pinquart M, Duberstein P (2002). How Effective Are Interventions With Caregivers? An Updated Meta-Analysis. The Gerontologist.

[9] Cameron JI, Naglie G, Green TL, Gignac MA, Bayley M, Huijbregts M, et al. A feasibility and pilot randomized controlled trial of the “Timing it Right Stroke Family Support Program”. Clin Rehabil. 2015;29(11):1129-40 10.1177/0269215514564897 25552525

[10] Christie D, Weigall D (1984). Social work effectiveness in two-year stroke survivors: a randomised controlled trial. Community Health Stud.

[11] Forster A, Young J (1996). Specialist nurse support for patients with stroke in the community: a randomised controlled trial. Br Med J.

[12] Dennis M, O’Rourke S, Slattery J, Staniforth T, Warlow C (1997). Evaluation of a stroke family care worker: results of a randomised controlled trial. Br Med J.

[13] Mant J, Carter J, Wade DT, Winner S (2000). Family support for stroke: a randomised controlled trial. Lancet.

[14] Glass TA, Berkman LF, Hiltunen EF, Furie K, Glymour MM, Fay ME, Ware J (2004). The Families In Recovery From Stroke Trial (FIRST): Primary Study Results. Psychosom Med.

[15] Goldberg G, Segal ME, Berk SN, Schall RR, Gershkoff AM (1997). Stroke Transition after Inpatient Rehabilitation. Top Stroke Rehabil.

[16] Tilling K, Coshall C, McKevitt C, Daneski K, Wolfe C (2005). A Family Support Organiser for Stroke Patients and Their Carers: A Randomised Controlled Trial. Cerebrovasc Dis.

[17] Lilley SA, Lincoln NB, Francis VM (2003). A qualitative study of stroke patients’ and carers’ perceptions of the stroke family support organizer service. Clin Rehabil.

[18] Clark MS, Rubenach S, Winsor A (2003). A randomized controlled trial of an education and counselling intervention for families after stroke. Clin Rehabil.

[19] Ellis G, Rodger J, McAlpine C, Langhorne P (2005). The impact of stroke nurse specialist input on risk factor modification: a randomised controlled trial. Age Ageing.

[20] Burton C, Gibbon B (2005). Expanding the role of the stroke nurse: a pragmatic clinical trial. J Adv Nurs.

[21] Boter H, Group ftHS (2004). Multicenter Randomized Controlled Trial of an Outreach Nursing Support Program for Recently Discharged Stroke Patients. Stroke.

[22] Egan M, Anderson S, McTaggart J. Community Navigation for Stroke Survivors and Their Care Partners: Description and Evaluation. Top Stroke Rehabil. 2010;17(3):183–190. 10.1310/tsr1703-183 20797962

[23] O’Mahony PG, Rodgers H, Thomson RG, Dobson R, James OF (1997). Satisfaction with information and advice received by stroke patients. Clin Rehabil.

[24] McKevitt C, Fudge N, Redfern J, Sheldenkar A, Crichton S, Rudd AR, Forster A, Young J, Nazareth I, Silver LE (2011). Self-Reported Long-Term Needs After Stroke. Stroke.

[25] Tyson SF, Burton L-J, McGovern A, Sharifi S (2014). Service users’ views of the assessment process in stroke rehabilitation. Clin Rehabil.

[26] Tistad M, Tham K, von Koch L, Ytterberg C (2012). Unfulfilled rehabilitation needs and dissatisfaction with care 12months after a stroke: an explorative observational study. BMC Neurol.

