# Supplementary material for: Social work after stroke: identifying demand for support by recording stroke patients’ and carers’ needs in different phases after stroke
Source: BMC Neurol. 2016 Jul 20;16:111. doi: 10.1186/s12883-016-0626-z (PMC4955160; doi:10.1186/s12883-016-0626-z)
Supplement: Additional file 1: — Recruitment and verbal consent. Description of recruitment as well as obtaining and documentation of consent. Data management. Description of data management and generation of the questionnaire. (DOCX 18 kb) [file 12883_2016_626_MOESM1_ESM.docx]

*Recruitment and verbal consent*

*Patients, caregivers and health care professionals contacting the SSP were asked to fill out the questionnaire with the social workers after having received the services for which they made contact with the SSP. Verbal consent was obtained from the participants (patients, caregivers or health care professionals) who made initial contact with the SSP, and was documented by the social workers in accordance with requirements of the Ethics Commission at the Charité University Medicine Berlin. When web-based inquiries were made, patients were asked to contact the SSP by phone to obtain verbal consent and complete the questionnaire.*

*Data management*

*Data management was performed by the group “Clinical Epidemiology and Health Services in Stroke (CEHRiS)” of Center for Stroke Research, Berlin*

*To ensure accuracy, the questionnaire was scanned using automated scanning software (Cardiff Teleform from electric paper). Accuracy of the dataset was further confirmed by data management checking for internal consistency (for example checking that only the predefined numbers occurred as answer categories). Additionally the social workers, who collected the data checked random samples (5%) of the data base for consistency with the original questionnaire.*

*The item content of the questionnaire and the different response categories for the domains of social work were developed based on product and service descriptions of the German Society for Social Work in the Health Care System [*[*1*](#_ENREF_1)*,* [*2*](#_ENREF_2)*] and expanded on the basis of topics that were specifically important to stroke related social work (supplementary table 1).*

*Supplementary table 1*

| *Categories based on the service description of the “German Society for Social Work in the Health Care System”* | *Categories based on stroke specific expanded service description for social work* |
| --- | --- |
|  |  |
| *Medical rehabilitation,* | *Therapeutic and preventive services* |
| *Services/help for return to working life (vocational advice)* | *Medical questions (e.g. finding a specialized out-patient practitioner)* |
| *Out-patient services* | *Nursing care* |
| *In-patient services* |  |
| *Questions around obtaining benefits (social law)* |  |
| *Partly residential services* |  |
| *Assistance with reintegration* |  |
| *Other (such as self-help groups)* |  |

*Subcategories of kinds of services provided were further outlined based on the service description and partly expanded based on the case groups in clinical social work developed by Brühl [*[*2*](#_ENREF_2)*], analogous to the medical diagnosis related groups (DRG) as a basis for reimbursement for hospital services. For example, the category “out-patient services” included “home adaptations” and specifically “changes around the house/housing space”, “emergency house calls” and “aids at home” as defined by Brühl.* *The Brühl case group system has been empirically validated [*[*2*](#_ENREF_2)*]. Additionally, one of the two social workers in our study, took part in work contributing to the original development of Brühl’s classification system.*

*The questionnaire started with some general introductory questions regarding age, gender of stroke patients and contact person, place of current residence of the stroke patient, origin of the stroke patient, time since the event, and how the contact person was referred to the Stroke Service Point before the details about the services provided were assessed.*

*Predefined response categories were given and the respondent had to choose one of them (forced choice technique: for example Domain: age categories: <45, 45-54, 55-64, 65-74 and 75-84, >85; Domain: therapeutic and preventive services, categories: Physiotherapy, Speech therapy, Neuropsychology, nutritional intervention etc.).*

*For each question the different responses were coded according to categories or “no response”. For example Domain: sex: male: 1, female: 2, no statement: 9.*

*Consistency, comprehension and completeness of the questionnaire was discussed with the social workers and outlined in an interdisciplinary meeting including occupational therapists and physiotherapists before study onset. During data collection all parts of the questionnaire were addressed with the patients. If patients/carers had difficulties understanding certain terms used by the social worker (e.g. Neuropsychology) these were explained. By including all the categories of social services available to patients, based on descriptions of general and stroke-specific service models and by using closed questions the questionnaire aimed to capture data on all services available to stroke patients in the German social system.*

*For analysis of the data, the services categories (which differ mainly because of different reimbursement schemes in the German social law system) were summarized under the “benefits”* *category. Additionally, details on home adaptations (such as changes around the house/housing space, emergency house calls and aids at home) or different out-patient nursing services (general home nursing care, palliative care, short term nursing care, family nursing care) were summarized for the analysis.*

*Prior to study onset the two social workers practiced performing the questionnaire to confirm length of time for delivery (practicability) and to clarify potential open questions. Furthermore, during a pilot phase including 55 patients, delivery methods were monitored and evaluated. The respondents were also asked to provide feedback on comprehension and relevance of content. Some minor changes, all referring to the introductory section of the questionnaire were included: instead of the date of the event, the time since the event was recorded, items regarding knowledge of the German language and resident permit status were removed. The questionnaire was delivered at the end of the consultation with the social worker, as service delivery was the primary focus of the consultation.*

*The intended duration of the study was two years.*

*Given that the questionnaire content was based on established social work principles and that data were collected by two trained social workers, with more than 20 years experience in clinical social work with stroke patients, data collection methods were considered to have face validity*

References

1. **Produkt- und Leistungsbeschreibung der Klinischen Sozialarbeit** In*.*: Deutsche Vereinigung für Sozialarbeit im Gesundheitswesen e.V. 2. ; 2007.

2. Brühl A: **Fallgruppen der Sozialarbeit (FdS) als Antwort auf die Einführung der diagnosis related groups in Akut-Krankenhäusern**: Nomos Verlagsgesellschaft (Baden-Baden) 2004.
