# Supplementary material for: Social work after stroke: identifying demand for support by recording stroke patients’ and carers’ needs in different phases after stroke
Source: BMC Neurol. 2016 Jul 20;16:111. doi: 10.1186/s12883-016-0626-z (PMC4955160; doi:10.1186/s12883-016-0626-z)
Supplement: Additional file 2: Table S1. — Categories for description of social work service. A comparison of general description for social work services in the health care system with a stroke specific expanded service description used to generate the questionnaire used in the current study. (DOCX 12 kb) [file 12883_2016_626_MOESM2_ESM.docx]

Supplementary table 1

| *Categories based on the service description of the “German Society for Social Work in the Health Care System”* | *Categories based on stroke specific expanded service description for social work* |
| --- | --- |
|  |  |
| *Medical rehabilitation,* | *Therapeutic and preventive services* |
| *Services/help for return to working life (vocational advice)* | *Medical questions (e.g. finding a specialized out-patient practitioner)* |
| *Out-patient services* | *Nursing care* |
| *In-patient services* |  |
| *Questions around obtaining benefits (social law)* |  |
| *Partly residential services* |  |
| *Assistance with reintegration* |  |
| *Other (such as self-help groups)* |  |
